# Neuronal Vulnerability of the Entorhinal Cortex to Tau Pathology in Alzheimer’s Disease

**DOI:** 10.3389/bjbs.2024.13169

**Published:** 2024-10-07

**Authors:** Simi Zhang, Chelsea Ann Crossley, Qi Yuan

**Affiliations:** Biomedical Sciences, Faculty of Medicine, Memorial University of Newfoundland, St. John’s, NL, Canada

**Keywords:** entorhinal cortex, tau pathology, Alzheimer’s disease, memory, early detection

## Abstract

This review delves into the entorhinal cortex (EC) as a central player in the pathogenesis of Alzheimer’s Disease (AD), emphasizing its role in the accumulation and propagation of tau pathology. It elucidates the multifaceted functions of the EC, encompassing memory formation, spatial navigation, and olfactory processing, while exploring how disruptions in these processes contribute to cognitive decline in AD. The review discusses the intricate interplay between tau pathology and EC vulnerability, highlighting how alterations in neuronal firing patterns and synaptic function within the EC exacerbate cognitive impairments. Furthermore, it elucidates how specific neuronal subtypes within the EC exhibit differential susceptibility to tau-induced damage, contributing to disease progression. Early detection methods, such as imaging techniques and assessments of EC blood flow, are examined as potential tools for identifying tau pathology in the preclinical stages of AD. These approaches offer promise for improving diagnostic accuracy and enabling timely intervention. Therapeutic strategies targeting tau pathology within the EC are explored, including the clearance of pathological tau aggregates and the inhibition of tau aggregation processes. By understanding the molecular and cellular mechanisms underlying EC vulnerability, researchers can develop more targeted and effective interventions to slow disease progression. The review underscores the importance of reliable biomarkers to assess disease progression and therapeutic efficacy in clinical trials targeting the EC. Ultimately, it aims to contribute to the development of more effective management strategies for AD, emphasizing the translation of research findings into clinical practice to address the growing societal burden of the disease.

## Introduction

Alzheimer’s disease (AD) is a neurodegenerative disorder that affects millions of people worldwide with an increasing prevalence [1]. It is estimated that over 10% of Americans above the age of 65 have AD and the number of AD patients could double by 2060 barring novel therapeutic approaches [2]. The management of AD is hindered by the absence of curative therapies and challenges in early detection, attributed to the delayed onset of symptoms subsequent to the formation of abnormal protein aggregates.

The entorhinal cortex (EC) emerges as a critical structure implicated in the accumulation and spread of tau pathology in AD [3–5]. Increasing evidence substantiates the EC’s role as the point of origin for the propagation of pathogenic tau [6]. To further establish the EC as a strategic focal point for early detection and therapeutic interventions, it is imperative to understand the underlying processes that render it particularly susceptible to tau pathology. In this review, we initially outline the distinct vulnerability of the EC, elucidating the molecular and physiological mechanisms at play, as well as the specific cell types within the EC that contribute to this vulnerability. Subsequently, we examine the clinical ramifications of these findings, exploring their potential implications for early detection strategies, the advancement of therapeutic interventions and the assessment of treatment efficacy.

## The Entorhinal Cortex (EC) as a Critical Structure in Tau Pathogenesis of Alzheimer’s Disease

### Tau Pathology Is Closely Associated With Alzheimer’s Disease (AD) Progression

**FIGURE 1:**
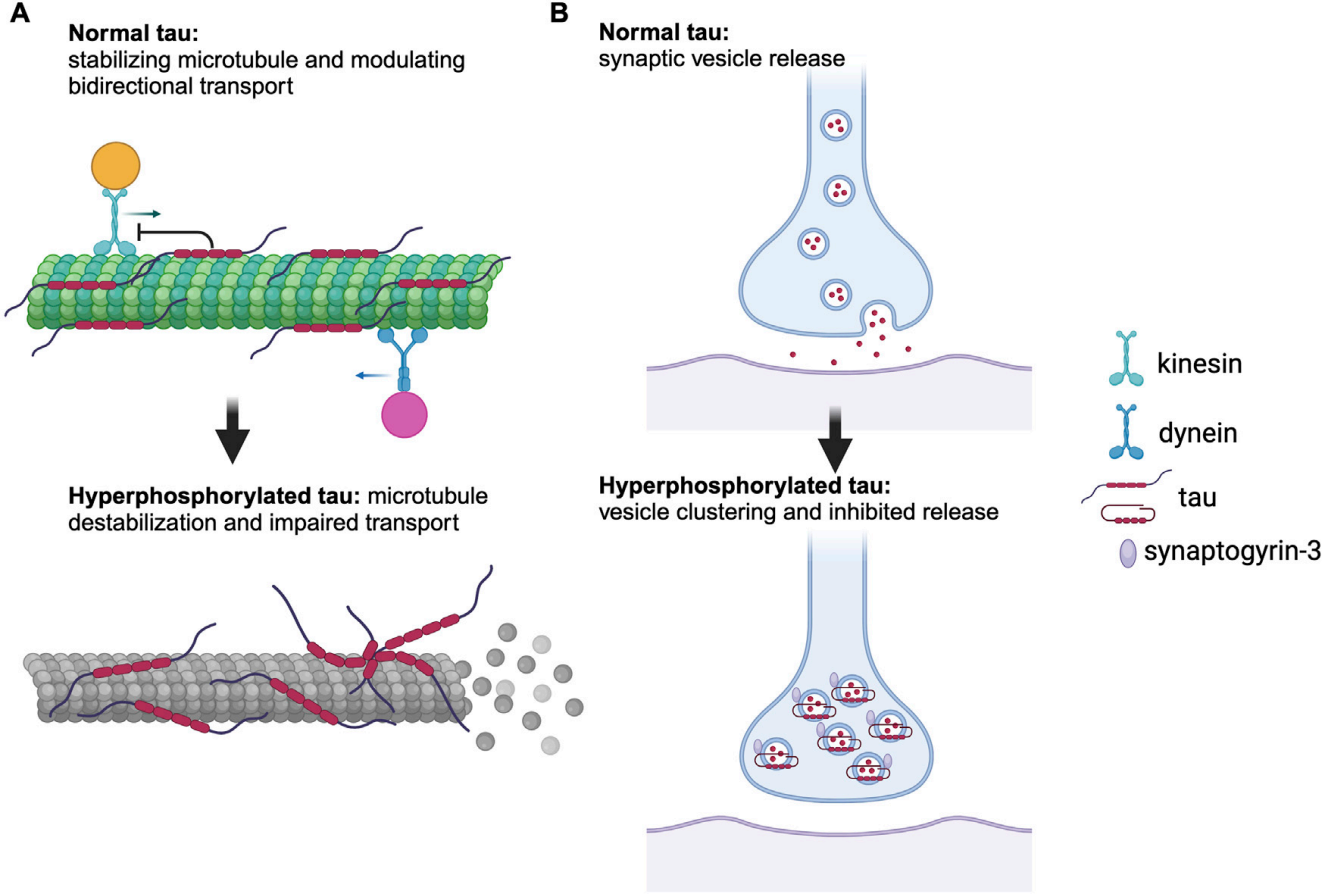
Role of normal and pathological tau in axonal transport and synaptic function. **(A)** Normal tau stabilizes microtubules and modulates bidirectional transport by preferentially inhibiting kinesin over dynein, whereas hyperphosphorylated tau causes microtubule disassembly and dissociation of microtubule-associated proteins, leading to impaired axonal transport. **(B)** Hyperphosphorylated tau interacts with synaptogyrin-3, resulting in vesicle clustering and inhibition of neurotransmitter release. Generated with BioRender.com.

Neurofibrillary tangles (NFTs) of tau proteins are one of the main sources of AD pathology. Tau protein is a microtubule-associated protein encoded by the MAPT gene on chromosome 17 [7]. It comes in six highly soluble isoforms produced by alternative splicing [8]. The longest isoform contains 85 possible phosphorylation sites, allowing for a high degree of heterogeneity in tau phosphorylation patterns in tauopathies [9]. The normal physiological role of tau is to induce tubulin assembly and stabilize microtubules, which are crucial for transporting molecules from the cell body of the neuron to the axon terminal [10]. Tau can take on two distinct forms while bound to microtubules – as independently diffusing molecules or in the form of cohesive envelopes. These cohesive envelopes can regulate interactions of the microtubule with other microtubule-associated proteins, such as kinesin and dynein, which migrate along the microtubule for anterograde and retrograde transport, respectively [11]. Normal tau acts as an obstacle to these motor proteins on the microtubule and modulates the balance of bidirectional transport by preferentially inhibiting kinesin over dynein (Figure 1A) [12]. Filamentous tau, however, is able to inhibit kinesin at normal physiological concentrations, thereby impairing anterograde transport [13]. Moreover, tau plays a role in synaptic vesicle control by interacting with synaptogyrin-3 [14]. Synaptogyrin-3 mediates the binding of pathological tau to synaptic vesicles, which leads to presynaptic vesicle clustering and inhibits neurotransmitter release (Figure 1B) [14]. In AD, tau undergoes a series of post-translational modifications that impact its function and contribute to disease progression. Phosphorylation is regulated in concert by kinases and phosphatases and any disruption of this balance may lead to tau hyperphosphorylation, favoring NFT formation [15]. Acetylation impairs tau-microtubule interaction and promotes tau aggregation [16]. Truncation contributes to apoptosis and tau aggregation [17]. Non-pathological tau contributes significantly to microtubule stability, whereas its post-translational modifications can destabilize the microtubules [18], thereby impairing axonal transport of molecules and organelles and consequently impairing neuronal function [19].

Accumulation of neurofibrillary tangles is one of the hallmarks of AD. Unlike amyloid plaques, which are present in subjects with no cognitive deficits and absent in some AD patients, hyperphosphorylated tau (p-tau) and NFTs correlate strongly with cognitive decline in AD patients [20]. Tau accumulation begins with the formation of pretangles, a soluble form of tau that exists at a higher density, often in monomers or dimers [21]. Pretangles also exhibit AT8 immunoreactivity but can be differentiated from mature tangles with a negative silver Gallyas staining [21]. Pretangles are classified into five stages (a, b, c, 1a, and 1b) based on the extent of their spread from the locus coeruleus (LC): pretangles in stages a, b, and c are more closely associated with the LC while those in 1a and 1b are marked by spread to the EC [21]. Hyperphosphorylation of tau results in its detachment from microtubules and self-assembly into insoluble aggregates [22]. These aggregates exist in two forms–paired helical filaments (PHFs) which are loosely intertwined and straight filaments (SFs) which are tightly wrapped [23, 24]. These two types of filaments further aggregate to form NFTs, which induces microtubule disassembly. This disruption in microtubule organization impairs axonal transport, resulting in synaptic dysfunction, neuronal loss, and reduced neural plasticity, which are critical pathological processes in AD [25].

### EC Is a Key Component of the Memory Circuitry That Is Primarily Affected by AD

Cognitive decline in AD initially surfaces as impairments to semantic memory, episodic memory, and visuospatial processing. These impairments manifest as difficulties in speech production, impaired encoding of recent events, and difficulties with spatial navigation [26]. As memory functions progressively decline, AD patients later present with impaired executive functions and sensorimotor symptoms which can impede their daily activities [27, 28].

**FIGURE 2:**
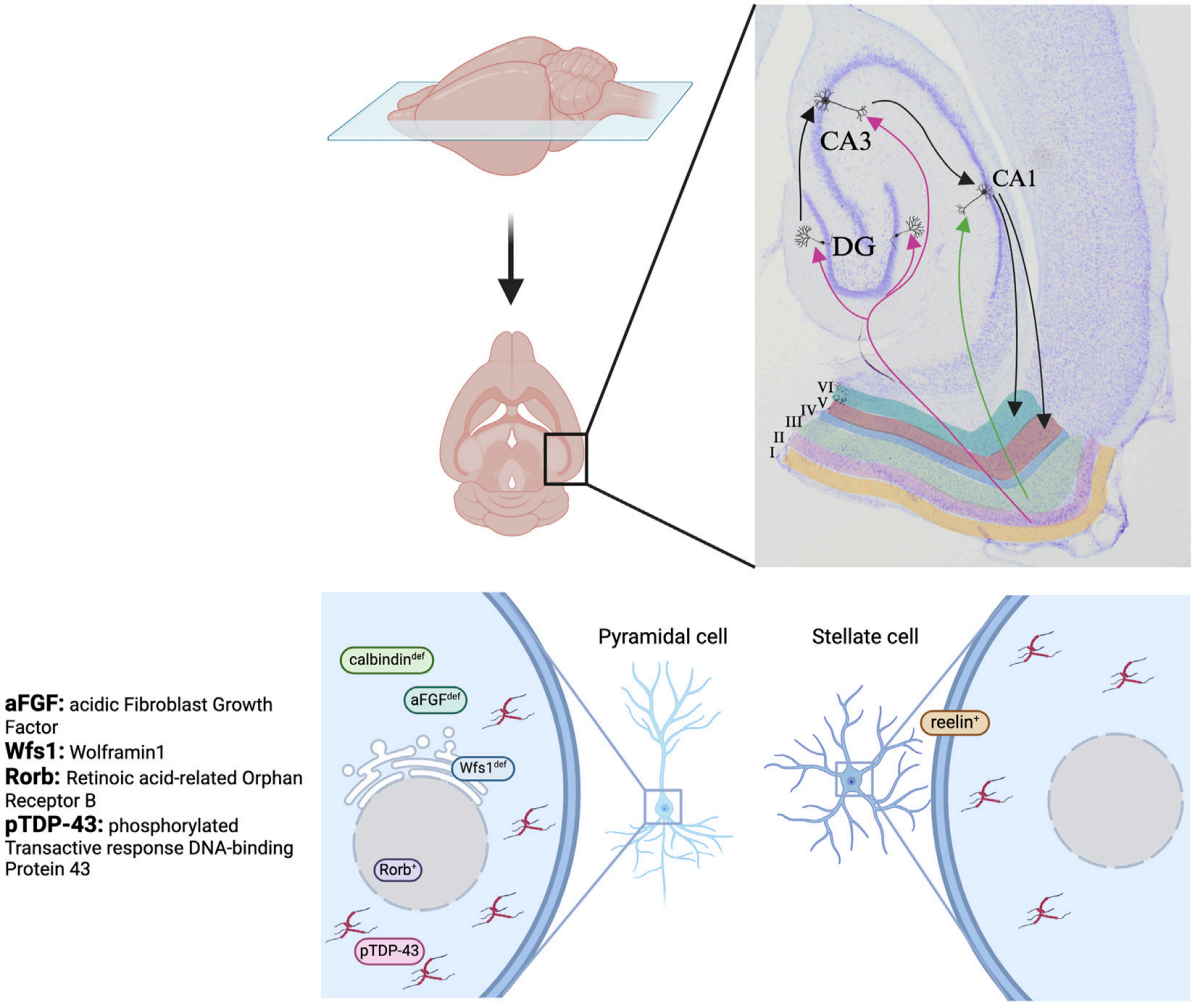
Cellular vulnerability of EC layer II neurons. These neurons project to the dentate gyrus (DG) and CA3, with subsequent relay to CA1. This diagram illustrates the specific molecular markers that favour tau accumulation in the two principal cell types in layer II of the EC, pyramidal cell (left) and stellate cell (right). Tau preferentially accumulates in pyramidal cells that are deficient in calbindin, aFGF, or Wfs1, or express Rorb and pTDP-43, and stellate cells that express reelin. Generated with BioRender.com.

Many aspects of these cognitive deficits in AD are mediated by the EC – a key component of the hippocampal network. The EC is located in the medial temporal lobe, partially enclosed by the rhinal sulcus which is involved in olfactory perception [29]. As part of the hippocampal formation, which also includes the hippocampus, the dentate gyrus (DG), and the subiculum, the EC plays a pivotal role in memory encoding and retrieval. Structurally, the EC is organized into six distinct layers, where layers I and IV are free of neurons (Figure 2) [29]. A number of subdivisions have been proposed for the EC, but it is most commonly divided into two anatomical regions, the medial entorhinal cortex (MEC) and the lateral entorhinal cortex (LEC) [30]. Despite their similar nomenclature, the MEC and the LEC have drastically different cytoarchitecture and connectivity. The MEC innervates the middle third of the DG whereas the LEC innervates the outer third. Their projections to the hippocampus also differ in that the MEC innervates proximal CA1 which has higher spatial specificity while the LEC innervates the distal CA1 [31]. Consequently, the MEC and the LEC are considered to be in two parallel processing streams into the hippocampus. Their connectivity dictates their function. The MEC is involved in processing spatial information and path integration while the LEC contributes to the processing of olfactory information [32]. These cognitive functions closely align with the initial presentations of deficits observed in AD, specifically impairments in spatial navigation and olfactory perception. Cells in the MEC are responsible for sensing the environment and the animal’s relative position in space. Subsequently, this spatial information is consolidated and transmitted via the perforant path, with layer II neurons of the MEC projecting to the DG and CA3 regions, while layer III neurons extend projections to the CA1 and CA2 regions of the hippocampus (Figure 2) [33]. The microcircuitry underlying olfactory processing initiates within the olfactory bulb, where odors are detected, and sensory information is transmitted to layer I of the LEC. Subsequently, this information is relayed to neurons residing in layers II and III of the LEC [26]. Here, the LEC integrates olfactory signals with contextual information before further relaying them to the CA1 and DG regions of the hippocampus. In both cases, CA1 and the subiculum send feedback to layers V and VI of the EC to refine the spatial and olfactory information. Given the crucial role of the EC in spatial and olfactory processing, the EC may be the key to detecting AD prior to the onset of cognitive deficits.

The EC is composed of chemically, morphologically, and functionally distinct cell populations. These cells can first be chemically distinguished as reelin- or calbindin-expressing based on the expression of reelin, a glycoprotein involved in cell-cell interactions and neuronal migration, or calbindin, a calcium-binding protein that protects neurons from Ca^2+^-induced damage [33]. These cells can be further divided into subgroups based on their morphology. In the MEC, neuronal populations consist of stellate cells and pyramidal cells, while in the LEC, they consist of fan cells, pyramidal cells, and multipolar cells. Stellate cells and fan cells within their respective regions express reelin, whereas pyramidal cells express calbindin [33]. Functionally, the MEC contains a variety of neurons involved in spatial coding, among which grid cells are most prominent in layer II and can exist as either stellate or pyramidal grid cells [34, 35]. In the following section, we will discuss the varying types of susceptibility of these cell types to tau pathology.

## Entorhinal Cortex Vulnerability

### Tau Pathology in the EC

According to the prion model of tau seeding, tau aggregates originating from one brain region have the capacity to escape the cells and serve as “seeds,” triggering the abnormal folding of tau proteins in other brain regions, prior to any observable histopathological changes [36]. Although the locus coeruleus (LC) is the site where abnormally phosphorylated pretangle tau is first detected, the prion-like seeding activity of abnormal tau first appears in the EC. This is demonstrated using a fluorescence resonance energy transfer (FRET) biosensor targeting the aggregation-prone repeat domain of tau [4]. The extensive connections linking the EC and other components of the hippocampal formation facilitate the transport and spread of NFTs. Using an epidemic spreading model derived from position emission tomography (PET) scans collected from AD patients across different disease stages, Vogel et al. [6] conclude that the network of anatomical connections can account for 70.2% of tau spatial localization. Furthermore, their model suggests that the EC is most likely the epicenter of tau propagation. Additionally, a rhesus monkey model of tau seeding demonstrates the spread of tau to many hippocampal subfields, encompassing CA3, CA1, the subiculum and dentate gyrus [37]. These insights collectively emphasize the pivotal role of the EC in tau seeding and propagation, prompting further exploration into the precise molecular mechanisms driving the EC’s vulnerability to tau pathology.

### Neuroinflammation in the EC

In addition to the abnormal aggregation of tau and β-amyloid proteins, chronic neuroinflammation is also a hallmark of AD. Deposition of insoluble NFTs is a classic stimulant for inflammation in the AD brain through the activation of reactive astrocytes and microglia, which subsequently release a wide range of proinflammatory molecules: cytokines such as IL-6, IL-1β, TNF-⍺, chemokines, reactive oxygen species (ROS), and nitric oxide (NO). These mediators in turn induce cellular damage and oxidative stress to neurons, leading to neurodegeneration [38]. Additionally, inflammation leads to the activation of various kinases, such as glycogen synthase kinase 3β (GSK3β) and cyclin-dependent kinase 5 (CDK5), which contribute to tau hyperphosphorylation, resulting in the formation of PHFs and NFTs [39]. In murine models, deficiency in the triggering receptor expressed on myeloid cells 2 (TREM2), a receptor vital for the survival and proliferation of microglia, has been demonstrated to diminish neuroinflammation and provide protection against volume loss in the EC [40]. Furthermore, elevated levels of soluble TREM2 in the cerebrospinal fluid (CSF), observed in symptomatic AD patients, are associated with neuroinflammation and p-tau accumulation [41]. Another proinflammatory mediator that contributes to neuroinflammation in the EC is the glia maturation factor (GMF). GMF is upregulated in regions where NFTs are present, leading to an increase in reactive astrocytes and activated microglia [42]. Additionally, evidence indicates that the administration of anti-GMF antibodies attenuates neuroinflammation and reverses behavioral deficits in a murine model. However, the direct impact of GMF blockade on NFTs remains unclear [43]. Collectively, these studies suggest the potential of TREM2 and GMF as biomarkers for monitoring neuroinflammation and neuronal loss in the EC.

### EC Hypoperfusion

EC hypoperfusion is another key pathogenic feature in early-stage AD. Impaired neurovascular coupling has recently been proposed as a possible mechanism of AD pathophysiology. The initial findings from the Alzheimer’s Disease Neuroimaging Initiative (ADNI) have identified an association between reduced cerebral blood flow (CBF) and tau accumulation in the EC [44]. Kapadia et al. [39] investigated the temporal relationship between these phenomena using a 6-year dataset from ADNI-2. They found that hypoperfusion in the EC precedes NFT deposition. Moreover, in the left EC, they observed a significant negative correlation between CBF and the severity of cognitive decline. Furthermore, CBF exhibited an inverse relationship with tau accumulation in the left EC over the 6-year period [45]. However, it is important to note that only one AD patient participated in the follow-up, necessitating further data to validate these findings and establish clinically relevant reference ranges for CBF in the EC. Nevertheless, these results highlight EC hypoperfusion as a potential predictor for tau presence in the EC, enabling early identification of individuals at risk of AD or in pre-MCI AD stages. These findings shed light on the role of cardiovascular diseases as risk factors for AD, emphasizing the importance of addressing these comorbidities to attenuate disease progression.

### EC Cellular Vulnerability to Tau

#### Reelin^+^ Stellate Cells in EC Layer II Are Preferentially Susceptible to Tau

Stellate cells are one of the principal cell types in layer II of the EC. In a mouse model, chronically silenced reelin^+^ stellate cells in EC layer II are outcompeted by active stellate cells in a circuit refinement process, leading to their degeneration [46]. Additionally, reelin^+^ stellate cells connecting the EC and the hippocampus are particularly vulnerable to such degeneration. Although this study did not demonstrate a direct link between pathological tau and neuronal silencing, in a mouse model, non-aggregated pathological tau proteins, lacking neurofibrillary tangles (NFTs), are capable of inducing neuronal activity suppression and subsequent neuronal silencing [47]. Further, in a murine model, stellate cells in the dorsal MEC exhibit an altered action potential firing pattern in the presence of mutant tau, which may contribute to the impaired spatial memory in AD [48]. Consequently, stellate cells within EC layer II warrant attention due to their vulnerability to neurodegeneration and alterations in electrical properties. Additionally, reelin levels in CSF correlate with tau levels [49], though further investigations are required to elucidate any potential correlation between CSF reelin levels and tau deposition within the EC.

#### Wolframin-1 (Wfs1^+^) Pyramidal Cells Propagate Tau From EC Layer II to the CA1

Pyramidal cells located in EC layer II expressing Wfs1 emerge as prime targets for tau accumulation. In this layer, Wfs1 strongly colocalizes with calbindin, consistent with the positive calbindin expression of pyramidal cells [50]. Wfs1, an endoplasmic reticulum (ER) transmembrane protein, regulates Ca^2+^ homeostasis and its downregulation can induce ER stress and apoptosis [51, 52]. Post-mortem studies have identified Wfs1^+^ pyramidal cells as pivotal contributors to tau propagation as they interface directly with CA1 pyramidal neurons, enabling a direct transfer of tau to CA1 dendrites [53]. Molecular and behavioral studies in murine models further confirmed tau propagation from Wfs1^+^ pyramidal cells in EC layer II to CA1, evidenced by disrupted CA1 neuron excitability and impaired contextual learning [53]. However, this study solely identifies the role of Wfs1+ pyramidal neurons in tau propagation, without elucidating the impact of Wfs1 levels on tau pathology. Subsequent research identified Wfs1’s interaction with tau at the ER and synapse in human AD brains. Moreover, overexpression of Wfs1 reduces tau seeding and aggregation, while its deficiency exacerbates these changes in murine models [54]. Additional studies are needed to examine whether Wfs1 deficiency directly leads to tau accumulation or if pathological tau overwhelms the protective effects of Wfs1. Such studies could position Wfs1 as an early biomarker of AD tau pathology.

#### Tau Tangles Accumulate Preferentially in Retinoic Acid-Related Orphan Receptor B (RORB)^+^ Excitatory Neurons

In AD, patients often experience challenges with ambulation, notably marked by difficulties in maintaining gait stability and balance [55]. Functionally, RORB acts as a transcription factor crucial for maintaining a fluid gait by regulating sensory feedback during ambulation [56]. A single-nucleus RNA-seq analysis of post-mortem human brains demonstrated that excitatory neurons expressing RORB are selectively vulnerable in AD, namely, that tau tangles preferentially accumulated in these neurons in the EC [57]. During AD progression, RORB^+^ excitatory neurons in the EC are gradually depleted, as demonstrated in the reduction of RORB^+^ neurons in higher Braak stages [57], making RORB a promising marker for early AD detection.

#### Transactive Response DNA-Binding Protein 43 (TDP-43) Colocalize With p-Tau in the EC

In addition to p-tau, TDP-43 is another common pathology in AD that shows a strong association with cognitive deficits and medial temporal atrophy [58]. Widely expressed in the central nervous system, TDP-43 is a regulator of RNA splicing and favors tau with four microtubule-binding repeats (4R-tau) by promoting inclusion of tau exon 10 [59]. Phosphorylated TDP-43 (pTDP-43) colocalizes with p-tau in the EC in post-mortem brains, though it is unclear if the two proteins interact and whether such interaction contributes to tau pathology in the EC [60]. In addition, plasma TDP-43 levels are also found to be negatively correlated with the volume of the EC [61]. Taken together, TDP-43’s strong association with cognitive deficits, p-tau, and EC volume suggest that it may serve as a potential therapeutic target and biomarker for AD.

#### Reduced Calbindin and aFGF Expression in Pyramidal Cells Increases Susceptibility to Neurodegeneration

In AD brains, pyramidal cells located in EC layer II show drastically reduced levels of calbindin and acidic fibroblast growth factor (aFGF) [62]. Calbindin and aFGF interact to play a major role in neuroprotective-neurotoxic balance by regulating calcium homeostasis. When downregulated, the neurons become more susceptible to neurodegeneration, contributing to the vulnerability of the EC [62]. No correlation was observed between the number of NFTs and calbindin or aFGF levels in the post-mortem brains. This is consistent with their neuroprotective properties, suggesting that the loss of EC layer II neurons expressing decreased levels of calbindin and aFGF may have occurred prior to death [62]. Further, it has been shown in rhesus macaque models that calbindin expression is prominent in EC layer III but absent in layer II [63, 64]. This may explain why EC layer II is most vulnerable to tau pathology, highlighting the key role of calbindin in the selective vulnerability of the EC.

#### Grid Cells Exhibit Reduced Firing and Periodicity in the Presence of Tau

Path integration, which is the ability to estimate one’s current position relative to a known starting point based on the body’s own movements rather than visual cues, is among the deficits that manifest early in AD [65]. During spatial navigation, grid cells from layer II of the MEC fire in a tessellating pattern as an animal moves through specific locations of its environment, creating a mental map to track its relative location in the space [66]. Human studies using virtual reality navigation have shown that larger errors in path integration in AD patients with MCI correlate with reduced EC volume and elevated cerebrospinal fluid (CSF) tau levels [67]. In aged mice burdened with tau accumulation, grid cells exhibit impaired specificity of grid field patterns, reduced peak and average firing rates, and lower information content compared to age-matched controls [68]. These molecular findings are further strengthened by behavioral studies which demonstrate that the presence of NFTs in the EC impairs path integration in mice [69]. This phenomenon strongly correlates with deficits evident in spatial memory tasks during the early stages of AD, underscoring the significance of impaired path integration as a potential avenue for early detection or prediction of AD. However, further studies in human are needed to confirm the link between the increased p-tau in EC grid cells and impaired path integration performance.

## Therapeutic Implications of the EC in AD

### Early Detection of Tau Pathology in the EC

#### Current Methods for Detecting Tau

Accumulation of abnormal tau in the EC is one of the first events in AD and precedes any memory deficits and cognitive decline. Given that current treatments for AD primarily address symptoms rather than provide a cure, early detection holds promise for enabling timely interventions aimed at slowing disease progression, particularly for individuals in preclinical stages. There are a number of tau PET tracers available, including ^18^F-MK-6240 [70], ^18^F-flortaucipir [71], ^18^F-RO-948 [72], and ^18^F-PI2620 [73]. Among these second-generation PET tracers, in 2020, ^18^F-flortaucipir (Tauvid) was the first to receive FDA approval for detecting NFTs in potential AD patients [74]. In a large-scale clinical trial evaluating the fidelity of ^18^F-flortaucipir, ^18^F-RO-948, and ^18^F-PI2620 in a number of regions of interest, including the EC, the three tau PET tracers have comparable diagnostic performance [75]. In another study involving the ^18^F-MK-6240 tracer [76], ^18^F-MK-6240 exhibits high accuracy in detecting tau deposition in the EC in both early and late stage AD. These tracers have made detection of EC tau deposition possible in living AD patients, which will facilitate the early detection and diagnosis of AD.

#### Potential Screening Method

The finding by Kapadia et al. [45] that reduced EC blood flow precedes tau deposition suggests a potential indirect approach to detect tau pathology in the EC. Implementing this technique in the clinical setting would entail monitoring for changes in EC CBF over the span of several years using arterial spin labelling (ASL) perfusion MRI. Unlike traditional tau-PET scans, ASL perfusion MRI is more accessible as it does not require the injection of tau tracers. Further, ASL perfusion MRI does not require significantly more resources than standard MRIs, only more scan time and data processing. Therefore, ASL perfusion MRI holds the potential to be integrated into routine screenings for patients with established AD risk factors.

### Therapeutic Strategies Targeting Tau Pathology

The early involvement of the EC in AD tau pathology underscores the clinical potential to combine tau clearance therapies with the use of early EC biomarkers. This approach aims to contain pathologic tau within the EC during the early stages of AD while closely monitoring the region to evaluate therapeutic efficacy. Currently, there is no cure available for tauopathy, but several classes of therapies aiming to halt its progression are in development. These therapies encompass traditional immunotherapies such as monoclonal antibodies against tau, tau vaccines, antisense oligonucleotides (ASO) targeting the MAPT gene, and inhibition of tau aggregation. Additionally, newer immunotherapies leverage mechanisms involving ubiquitin, autophagy, and lysosome for tau degradation [77]. For an extensive review of current tau-targeting therapies for AD, Congdon et al. [78] have meticulously outlined the mechanisms of action for each category of tau-targeting therapy, along with the current clinical trial status of these interventions. In this section, our focus lies primarily on tau clearance strategies in early AD and the monitoring of disease progression through biomarkers associated with EC tau pathology.

#### Tau Passive Immunotherapy

Passive immunotherapy utilizes anti-tau antibodies to specifically target abnormal tau without affecting its normal function in stabilizing microtubules. The most recent advances in antibodies targeting abnormal tau are summarized in Table 1. Since intravenous administration of the antibodies does not allow for specific targeting of the EC, it is crucial to select candidates with a high therapeutic index. This approach ensures that in early-stage AD prior to the spread of pathologic tau from the EC, abnormal tau can be effectively cleared while minimizing potential toxicity in other unaffected regions of the brain.

**TABLE 1:** Current anti-tau antibodies.

Anti-tau antibody	Mechanism of action	Phase	Clinical trial	n	Target population	Completion	Reference
E2814	Prevents tau spread by binding to the HVPGG epitope in the microtubule-binding region near the mid-domain of tau	I/II II/III	NCT04971733 NCT05269394	8 168	Moderate AD with APP or PSEN mutations	2025–07 2027–10	[79]
Semorinemab	Targets all known isoforms of full-length tau protein by binding to the N-terminal of tau (aa 6–23)	II	NCT03828747	238	Mild-to-moderate AD	2023–08	[80, 81]
APNmAb005	Prevents tau propagation by binding preferentially to tau oligomers at synapses	I	NCT05344989	40	Healthy patients	2024–07	[90]
Bepranemab	Reduces levels of pathologic tau by targeting mid-region of tau	II	NCT04867616	421	Prodromal or mild AD	2025–07	[82]
JNJ-63733657	Neutralizes tau seeds and inhibits propagation by binding to MTBR, specifically residue 217 of p-tau	II	NCT04619420	421	Early AD	2032–12	[83]
PNT001	Recognizes cis p-tau at residue 231	I	NCT04096287	49	Healthy patients	2021–02	[84]
Lu AF87908	Binds pS396-tau to reduce levels of p-tau and tau aggregates	I	NCT04149860	86	Healthy patients and mild AD	2023–07	[85]
MK-2214	Targets p-tau at Ser413	I	NCT05466422	48	MCI or mild-to-moderate AD	2025–05	[86]

##### E2814

E2814 is an anti-MTBR tau antibody that targets the HVPGG epitope in the microtubule-binding region (MTBR) near the mid-domain of tau. The preclinical *in vivo* studies on E2814 exhibited specific binding to all forms of tau in post-mortem AD brain and the murine version of E2814 attenuated the deposition of sarkosyl-insoluble tau [79]. There are two clinical trials ongoing for E2814, NCT04971733 in Phase I/II and NCT05269394 in Phase II/III which combines E2814 and the anti-Aβ antibody lecanemab.

##### Semorinemab

Semorinemab (RO7105705, MTAU9937A, RG6100) is a humanized IgG4 monoclonal antibody that targets all known isoforms of full-length tau protein by binding to the N-terminal of tau (aa residues 6–23). The Phase II clinical trial (NCT03828747), which was completed in August 2023, contained 238 patients with mild-to-moderate AD. After 49 weeks, Semorinemab showed a 42.2% reduction in the rate of cognitive decline on the 11-item Alzheimer’s Disease Assessment Scale-Cognitive Subscale (ADAS-Cog11) but no significant effect on the Alzheimer’s Disease Cooperative Study–Activities of Daily Living (ADCS-ADL) scale. These results suggest that Semorinemab contributes to cognitive but not functional improvements [80, 81]. The clinical efficacy of Semorinemab is demonstrated in the elevated plasma tau levels indicative of peripheral tau binding and domain analyses showing Semorinemab’s activity in the memory domain.

##### APNmAb005

APNmAb005 is a humanized IgG4 monoclonal antibody that binds to tau oligomers and aggregates, preferentially pathological tau in early Braak stage AD brains. The antibody is currently undergoing Phase I trial (NCT05344989) with 8 subjects. APNmAb005 reacts preferentially with tau proteins carrying three or four microtubule repeats (3R or 4R), as well as early-stage oligomers of tau enriched at synapses [87]. In the rTg4510 murine model, mAb005 blocked intracellular GFP-tau aggregation and partially rescued neuronal loss in the DG and CA1 [87]. Despite the preliminary nature of the evidence, APNmAb005 shows promising therapeutic effects for early-stage pathological tau and protective properties against neuronal loss.

##### Bepranemab

Bepranemab (UCB0107, UCB 0107, and Antibody D) is a humanized IgG4 monoclonal antibody targeting aa residues 235–250 in the mid-region of tau, near the MTBR [82]. A Phase II trial (NCT04867616) targeting MCI or mild AD is currently underway for Bepranemab. Preclinical studies in murine models show that Bepranemab reduces tau seeding induced by extracellular human pathological tau species and blocks tau propagation [82, 88]. These findings suggest that Bepranemab shows promise in halting the progression of AD in early stages of the disease, making it a viable therapeutic candidate to complement early detection of AD by monitoring changes in the EC.

#### Tau Aggregation Inhibitors

Considering that tau spreads from the EC to other regions of the brain in a prion-like manner, preventing tau propagation requires inhibition of abnormal tau aggregation. Several agents aligned with this approach are currently being evaluated.

##### Curcumin

Curcumin is a supplement derived from turmeric that has been demonstrated to reduce soluble tau dimers and elevate heat shock protein levels involved in tau clearance [89]. Although curcumin did not directly suppress levels of soluble p-tau monomers or insoluble tau, it did restore synaptic and behavioural deficits [89]. In another clinical trial examining the effect of curcumin in non-demented adults, Small et al. [90] found a significant difference in FDDNP-PET binding levels in the amygdala and hypothalamus in the curcumin group compared to the placebo. However, the study did not find a significant change in tau levels in the EC, which calls for further investigation on the efficacy of curcumin supplementation as a preventive treatment to reduce tau levels in pre-MCI AD patients. Given the safety, wide availability, and low cost, curcumin may serve as a promising preventative option if proven to be efficacious at reducing EC tau levels and cognitive deficits.

##### Methylene Blue

Methylene blue (TRx0237, LTMX) is being investigated for several tauopathies. Orally administered methylene blue has been demonstrated to reduce detergent-insoluble p-tau in P301L tau mice [91]. Furthermore, preventive methylene blue treatment administered prior to the onset of functional deficits, preserves cognition and decrease insoluble tau, as demonstrated in a mouse model expressing pro-aggregant human tau [92]. While the initial clinical trial where participants were permitted to take other AD medications failed to demonstrate benefits of LMTX [93], analysis of participants who were taking only LMTX exhibited significantly reduced AD progression. This suggests the possible therapeutic effects of LMTX as a monotherapy. A phase III trial (NCT03446001) of LMTX therapy in participants with probable or MCI AD was completed in April 2023, pending publication of results.

### EC Markers for Evaluating Treatment Benefits

Although the cost-effectiveness and feasibility of large-scale screening for AD biomarkers in the EC may be limited, these markers may be invaluable in assessing treatment benefits. A halt in disease progression may be signified by a decrease in CSF levels of TREM2, a marker of neurodegeneration and EC volume loss, or preservation of RORB^+^ excitatory neurons. CSF reelin levels have been highlighted as a proxy for tau levels but further research is needed to evaluate any correlation with tau pathology specifically in the EC [49]. Investigating the relationship between these potential biomarkers and AD tauopathy progression, would allow for more efficient and less resource-intensive methods to assess the benefits of AD therapies.

## Conclusion

### Summary of Key Findings

The EC occupies a central position in AD pathology and cognitive impairment. Its significance extends beyond merely being the site of initial tau aggregation, as it also plays crucial roles in learning and memory processes, particularly in spatial cognition and olfactory learning.

The EC’s intricate connectivity with key memory-related structures like the hippocampus and neocortex underscores its involvement in memory deficits observed in AD. The detrimental effects of tau pathology in the EC manifest in various ways, affecting grid cells' firing patterns, altering electrical gradients in stellate cells, and accelerating the degeneration of specific neuronal populations, such as reelin+ stellate cells. Specific neuronal subtypes within the EC, such as RORB-expressing excitatory neurons and Wsf1-expressing pyramidal cells, are selectively vulnerable to tau pathology. Additionally, reduced levels of neuroprotective factors such as calbindin in EC layer II render it particularly susceptible to tau-induced damage. Furthermore, the presence of NFTs in the EC triggers microglial activation and loss of pyramidal cells, contributing to sustained neuroinflammation.

### Implications for Future Research

Understanding the cellular, physiological, and molecular characteristics of the EC not only sheds light on the mechanisms underlying AD progression but also presents opportunities for early detection of tau pathology in this region. Consequently, further research into therapeutic interventions targeting the EC is warranted to develop effective treatments for AD.

Indeed, while various vulnerabilities to tau pathology have been identified in the EC, further research is crucial to elucidate the exact roles of the markers or proteins involved. This deeper understanding is essential for translating these findings into effective therapeutic strategies for AD.

Since slowing disease progression is the primary therapeutic goal for AD, enhancing tools for early detection of NFTs in the EC is critical. Research efforts should focus on evaluating the accuracy and specificity of tau tracers specifically for the EC, as well as exploring blood or CSF AD biomarkers that reflect the histological changes observed in this brain region.

Once more reliable and cost-effective detection methods are established, it will be possible to assess the efficacy and safety of AD therapies in individuals at preclinical stages. Targeting tau pathology in the EC before cognitive deficits manifest could potentially slow disease progression and improve outcomes for individuals at risk of developing AD.
